# Total thyroidectomy for giant nodular goiter guided by pre-operative 3D computed tomography reconstruction and 3D printing: A case report

**DOI:** 10.1097/MD.0000000000032456

**Published:** 2022-12-30

**Authors:** Jun Zhang, Wanli Liu, Qi Zhang, Chongru Zhao, Jie Li, Xing Li, Gezi Li, Jiali Chen, Dawei Peng, Yifei Wang, Chang Yang

**Affiliations:** a Department of Thyroid and Breast Surgery, Shenzhen Qianhai Shekou Free Trade Zone Hospital, Shenzhen, China; b Department of Plastic and Cosmetic Surgery, Tongji Hospital, Tongji Medical College, Huazhong University of Science and Technology, Wuhan, China.

**Keywords:** Complication, computer-assisted 3-dimensional imaging, giant nodular goiter, 3-dimensional printing, thyroidectomy

## Abstract

Nodular goiter is a common clinical problem, and thyroidectomy is preferred in patients with obstructive symptoms. Thyroidectomy is a complex procedure with some common complications. Three-dimensional (3D) computed tomography (CT) reconstruction and 3D printing provide visualized 3D anatomical structure, posing an enormously valuable potential in precise surgery with optimal efficacy and minimum complications. Here, we aimed to perform a precise thyroidectomy guided by this technology.

**Methods:**

The patient was an 80-year-old woman with 10 years of goiter, 1 year of labored dyspnea, and a history of thyroid surgery 62 years ago. In addition to ultrasonography examination, CT images were obtained to construct the 3D model to identify the 3D relationship between the lesion and adjacent structures, and a 3D model of the trachea was created and printed using a 3D printer.

**Results:**

The 3D model clearly presented the diffuse enlargement of the two lobes and isthmus and the compression of the goiter. Under the 3D guidance, the operative resection specimen of the right lobe and isthmus was 12 ´ 7 ´ 5 cm, whereas the left lobe specimen was 12 ´ 9 ´ 6 cm. Nodular goiter and lymphocytic thyroiditis were confirmed by postoperative histopathology. There were no complications after total thyroidectomy except for non-permanent hypocalcemia and hypoparathyroidism.

**Conclusion:**

Our results proved that total thyroidectomy for giant goiter is challenging, and 3D image-guided thyroidectomy facilitates precise and safe resection with fewer complications. 3D CT reconstruction and 3D printing can provide anatomical details and may be considered in thyroidectomy planning for patients with giant goiter.

## 1. Introduction

The thyroid consists of 2 lobes connected by the isthmus, located at the front of the neck below the thyroid cartilage, bordered by the trachea and esophagus posteriorly, and by the carotid sheath laterally. The volume and weight of the thyroid gland in healthy adults without iodine deficiency are 7 to 15 mL and 10 to 20 g, respectively.^[1–3]^ The abnormal enlargement of the thyroid gland is referred to as goiter.^[4]^ Goiter affects more than a tenth of the world’s population to some extent. Various treatment modalities are available for nontoxic nodular goiter, including observation, radioiodine, and surgery.^[5–7]^ Thyroidectomy is preferred for patients with obstructive symptoms, cosmetic concerns, substernal goiters, and suspected malignancy.^[8]^ Thyroidectomy is generally a safe procedure with a relatively low mortality rate. However, some postoperative complications still exist after thyroidectomy, including recurrent laryngeal nerves (RLNs) and trachea injury, hypocalcemia, and hematoma.^[8]^ Reducing postoperative complications requires consideration of many factors, including improved detection techniques and improved surgical procedures.

In addition, adequate preoperative assessment and planning also play an important role in preventing postoperative complications.^[9]^ Imaging examinations including computed tomography (CT) and magnetic resonance imaging are used to assess the extent of the goiter and its effect on surrounding structures, which are critical to ensure optimal surgical outcomes. However, traditional two-dimensional (2D) imaging alone may not provide an unequivocal and visualized assessment of the lesion and its surrounding tissues and blood supply. At present, three-dimensional (3D) reconstruction and 3D printing encourage a better understanding of complex anatomical details in complex pathological conditions and are being applied in multiple surgical fields, including neurosurgery, spine surgery, pelvic surgery, and liver surgery.^[10]^ The usefulness of this technique in thyroid surgery has been seldom reported and primarily focuses on preoperative education and obtaining an informed consent process.^[11,12]^ To our knowledge, the usefulness of 3D CT reconstruction and 3D printing to guide thyroidectomy has not been previously reported. In this case report, we reported that the 3D CT and 3D printing were used to precisely guide thyroidectomy for a patient who underwent total thyroidectomy and the patient made a good postoperative recovery.

## 2. Patient case presentation

**Figure 1.**
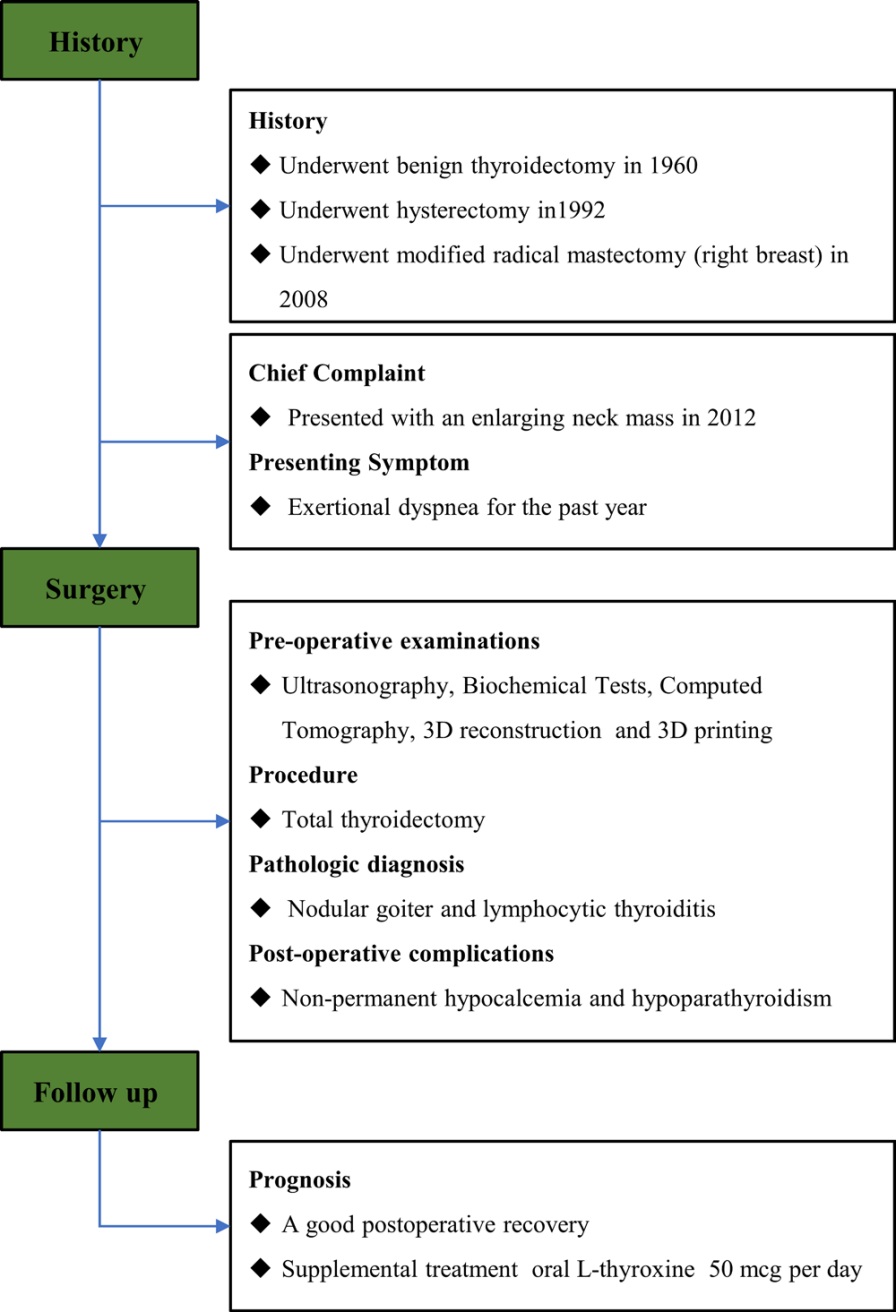
The flow chart of the history, preoperative preparation, surgical approaches, and follow-up of the patient.

An 80-year-old female presented with an enlarging neck mass for 10 years and exertional dyspnea over the past year. She underwent benign thyroidectomy at age 18, hysterectomy at age 50, and modified radical mastectomy at age 66. Additionally, she had hypertension for 10 years with well-controlled blood pressure with medications (Fig. 1). Biochemical tests revealed that the concentration of the hormones thyroxine (T4) and triiodothyronine (T3) was within the normal range, and serum thyroid-stimulating hormone was also within the normal level. However, thyroid peroxidase antibodies were tremendously elevated with a concentration of > 1006 IU/mL (reference range, 0.00–9.0 IU/mL).

**Figure 2.**
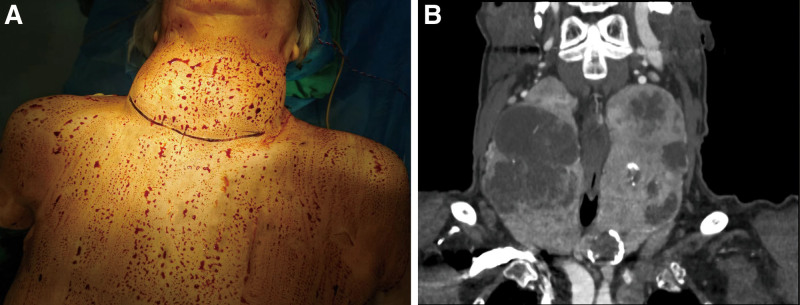
The preoperative findings. (A) Inspection: thy thyroid gland enlarged diffusely; (B) Coronal section of computed tomography showed diffuse enlargement of the 2 lobes of the gland.

**Figure 3.**
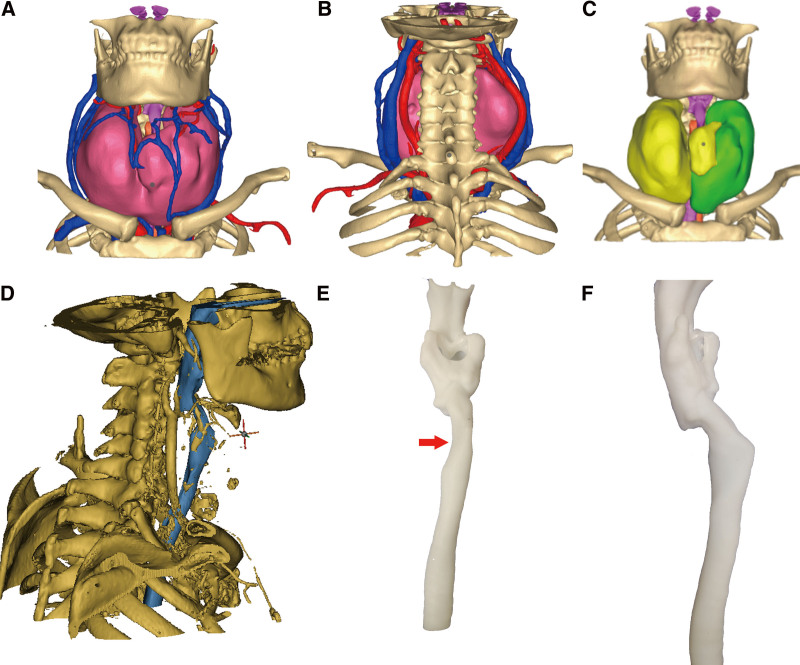
Three-dimensional reconstruction model and 3D printing model. (A–C) The enlarged gland and its relationship to adjacent structures. The carotid sheath was posterolateral bulged and has no communication with the lesion; (D, E) 3D model of the trachea and the compression of the trachea, as indicated by the arrow.

The patients underwent a neck and chest CT scan with a 64-slice dual-source CT (Siemens Healthcare, China). Diffuse enlargement of the thyroid gland and calcifications inside were detected, with the upper and lower boundary reaching the submandibular region and mediastinum, respectively. The CT scan showed that the right lobe size of the thyroid gland was 6.1 cm in depth, 6.1 cm in width, and 11.7 cm in length, and the size of the left lobe was 5.3 cm in depth, 4.2 cm in width, and 11.4 cm in length (Fig. 2). Moreover, the CT scan revealed the deviation of the trachea to the left side of the neck because of the compression of the giant goiter. To visualize the goiter and its surrounding tissues and blood supply, images were obtained to construct the 3D model to identify the 3D relationship between the lesion and adjacent structures (Fig. 3A–C). The 3D model showed the enlargement of the 2 lobes and isthmus. As a result, bilateral internal jugular vein and common carotid artery were posterolateral bulged (Fig. 3A and B). 2D CT images showed there was a compression of the trachea. To assess the position and degree of pressure on the trachea, we made a 3D model of the trachea and printed it using a 3D printer, thus providing supplemental information for the anesthesiologist to manage the airway (Fig. 3D–F). The 3D model of the trachea presented that the thyroid cartilage was compressed and flattened by an enlarged thyroid gland (Fig. 3E).

**Figure 4.**
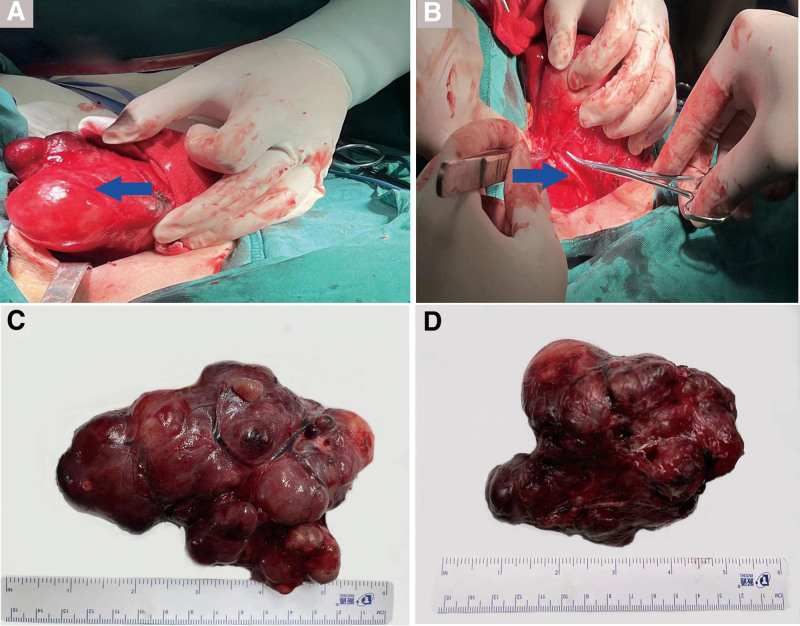
The dissection of the thyroid gland and operative resection specimen. (A) Lateral dissection of the right lobe of the thyroid gland, (B) Recurrent laryngeal nerve seen in the right tracheoesophageal groove, (C) Right lobe and isthmus (12 × 7 × 5 cm). (D) Left lobe (12 × 9 × 6 cm).

After careful routine examination and the anatomical details provided by 3D printing, the decision was made to perform a total thyroidectomy guided by this comprehensive preoperative evaluation. The patient was intubated and received general anesthesia during the thyroidectomy. In addition, intraoperative nerve monitoring was used to reduce the incidence of RLN injury. A transverse incision of approximately 8 cm was made via a previous surgical incision. After stripping the band muscle, it was found that the thyroid capsule was intact, the thyroid gland was enlarged, and the carotid sheath was compressed, which was consistent with the preoperative 3D model detection results (Fig. 4A). Total thyroidectomy was then performed and the gland was successfully dissected with high accuracy. The bilateral RLN was identified and protected using intraoperative nerve monitoring (Fig. 4B). First, the right lobe and isthmus of 12 × 7 × 5 cm were removed, considering that the gland obscured the detection of the other lobe (Fig. 4C). Subsequently, the left lobe of 12 × 9 × 6 cm. was successfully resected (Fig. 4D).

There were no clinical manifestations of dysphagia and hoarseness after surgery. However, hypocalcemia occurred on the first postoperative day with a calcium ion concentration of 2.06 mmol/L, but without symptoms of paraesthesia and tetany. The blood calcium levels returned to normal after calcium supplements with vitamin D on day 3 after surgery. The concentration of parathyroid hormone decreased to 3.66 pg/mL on postoperative day 1 and returned to normal with a concentration of 9.17 pg/mL on postoperative day 7. Pathologic examination revealed that nodular goiter was accompanied by cysts and hemorrhage, and that focal lesions were associated with infarction, necrosis, and calcification. Furthermore, lymphocytic thyroiditis was also confirmed by pathological evaluation. The patient received thyroid hormone replacement therapy with oral levothyroxine at 50 mcg/d. This patient recovered well post-operation and was discharged one week after surgery.

## 3. Discussion

Worldwide, iodine deficiency is the most common cause of nodular goiter in iodine-deficient regions.^[13]^ In addition, in areas with less severe iodine deficiency, goiter is most commonly caused by Hashimoto’s thyroiditis or Graves’ disease. In this case, chronic autoimmune thyroiditis (Hashimoto’s thyroiditis) resulted in the abnormal growth of the thyroid gland. Because the thyroid gland is in a position covered only by thin cervical muscles, the enlarged thyroid lobes usually grow outward, resulting in a large goiter that may not compress the trachea.

However, for patients with an asymmetric expansion of the thyroid gland, surrounding structures, including the trachea, esophagus, or blood vessels, may be displaced or less compressed. The goiter may be associated with normal, decreased, or increased thyroid hormone production.^[14]^ The management of goiter depends upon the cause and clinical manifestation. Surgical indications for goiter included patients with obstructive signs and symptoms, cosmetic concerns, or suspicion of malignancy.^[8]^ Total or near-total thyroidectomy or subtotal thyroidectomy may be considered for patients with giant goiter.^[15]^ Total or near-total thyroidectomy is preferred over subtotal thyroidectomy. A meta-review of 4 randomized trials reported that goiter recurrence was lower in the total thyroidectomy group compared to the subtotal thyroidectomy group.^[16]^ Surgery-related complications include wound hematoma, hoarseness, or change in voice due to nerve injury, hypocalcemia, hypoparathyroidism, or esophageal or tracheal injury. These situations compromise the health-related quality of life.^[17]^ Sufficient preoperative preparation and standard operation procedure are essential to prevent surgery-related complications. Thyroid imaging comprises ultrasonography, thyroid scintigraphy, CT, and magnetic resonance imaging.^[8]^ Any patient with thyroid nodules needs an ultrasound to assess their size, nodule location and adjacent structures, and cervical lymph nodes. It has been reported that the performance of preoperative cervical ultrasound is related to a lower incidence of hypocalcemia.^[9]^ However, neither ultrasonography nor CT could provide visualized stereo-image for the surgeon. With the application of 3D reconstruction and 3D printing in the medical field, it has become an effective tool in surgical planning and intraoperative guidance.^[18]^ 3D CT reconstruction and 3D printing have seldom been used in thyroid surgery.^[19]^

In this case, we used CT images to construct a 3D model of the thyroid gland and its surrounding structures and printed the trachea. The enlargement of the bilateral lobe caused compression of the trachea and jugular veins. Furthermore, there was no evidence that the goiter invaded the surrounding tissues, including the great vessels of the neck, which provided guidance in making surgical planning. Using a 3D model, we preoperatively evaluated the anatomic extent of the goiter and successfully and completely dissected the goiter. Additionally, the lateral edge of the lobe was bluntly dissected in the intraoperative period with minimal bleeding. The 3D printed model of the trachea showed the compression location, which could be considered during the process of intubation. In addition, the compressed position should be carefully checked after the removal of the thyroid gland. Trachea suspension should be performed when tracheal collapse occurs.

3D printing is well applied in surgical practice. Its applications vary from anatomical models to surgical guides and implants.^[20]^ For thyroid disease, the usefulness of 3D printing techniques was mainly reported in Graves’ orbitopathy to evaluate the proptosis.^[11]^ Furthermore, the clinical applications of the 3D-printed model in thyroid surgery largely focused on improving patients’ cognition and satisfaction during the process of patient education and informed consent.^[12,19]^ In our case, however, we focused primarily on making pre-operative planning and guiding resection of the thyroid gland. The application of 3D printing has not been previously reported in patients with giant goiter. For this patient, many risk factors may increase surgical risk, including advanced age, giant goiter, and thyroid reoperation. However, with the help of the 3D reconstruction model, we successfully performed a total thyroidectomy for this patient.

Although the unparalleled advantages of 3D CT reconstruction and 3D printing in preoperative planning have been successfully documented, there are some limitations in this study. First of all, there is only one case of surgery guided by this technology in this study, which is a limitation that we first acknowledge. Secondly, any mistake in each stage of a model establishment may result in inaccuracy, so it requires high technical requirements and strives to accurately present the diseased lesions. Furthermore, the entire process of 3D printing is technically demanding, time-consuming, and expensive, which may limit its use in large-scale clinical cohorts. Finally, the limitations of 3D CT reconstruction itself may prevent the construction of fine anatomical structures, such as the RLN.

## 4. Conclusion

The technique of 3D CT reconstruction and 3D printing can provide the anatomical details of the thyroid gland and surrounding tissue, thus possessing the ability of precise image-guided surgery for thyroidectomy. We carried out a total thyroidectomy for giant nodular goiter guided by pre-operative 3D CT reconstruction and 3D printing. These results emphasized that this technique poses an enormously valuable potential in precise surgery with optimal efficacy and minimum complications. It may be an option considered in surgical planning and intraoperative guidance for patients with giant goiter. Further studies are needed to determine the potential value of 3D printing in the preoperative planning process to formulate optimal surgical procedures.

## Author contributions

All authors have reviewed the manuscript and all approved of the final version.

**Data curation:** Xing Li, Gezi Li.

**Methodology:** Chang Yang.

**Project administration:** Jun Zhang.

**Software:** Dawei Peng.

**Supervision:** Jie Li, Jun Zhang, Qi Zhang.

**Validation:** Jiali Chen, Yifei Wang.

**Writing—original draft:** Wanli Liu.

**Writing—review and editing:** Chongru Zhao.
